# MCC950‐Loaded M12‐Liposome Nanoparticles for Targeted Inhibition of NLRP3 Inflammasome in Sepsis‐Induced Muscle Atrophy

**DOI:** 10.1002/jcsm.70285

**Published:** 2026-04-15

**Authors:** Yukun Liu, Kang Wang, Zhikai Xu, Xuan Zhao, Zhanfei Li, Xiangjun Bai, Hao Zhu, Fangli Gao, Liang Zhu, Yuchang Wang

**Affiliations:** ^1^ Department of Plastic and Aesthetic Surgery, Tongji Hospital, Tongji Medical College Huazhong University of Science and Technology Wuhan China; ^2^ Division of Trauma Surgery, Emergency Surgery & Surgical Critical, Tongji Hospital, Tongji Medical College Huazhong University of Science and Technology Wuhan China; ^3^ Trauma Center, Tongji Hospital, Tongji Medical College Huazhong University of Science and Technology Wuhan China; ^4^ Department of Emergency and Critical Care Medicine, Tongji Hospital, Tongji Medical College Huazhong University of Science and Technology Wuhan China; ^5^ Sino‐German Research Institute of Disaster Medicine Wuhan China; ^6^ Department of Orthopedic Surgery, Tongji Hospital, Tongji Medical College Huazhong University of Science and Technology Wuhan China; ^7^ School of Chemistry and Chemical Engineering Henan Normal University Xinxiang China

**Keywords:** liposome, MCC950, muscle atrophy, NLRP3, pyroptosis, sepsis

## Abstract

**Background:**

Sepsis‐induced myopathy (SIM) is a severe complication that contributes to late‐stage mortality and functional impairment in sepsis patients. The NLRP3 inflammasome plays a pivotal role in the pathogenesis of SIM, and its selective inhibitor MCC950 has shown promising therapeutic potential. However, systemic administration of MCC950 is limited by hepatotoxicity, necessitating the development of targeted delivery systems to enhance efficacy while minimizing toxicity.

**Methods:**

To improve the therapeutic profile of MCC950, we designed M12‐functionalized liposomal nanoparticles (M12‐Liposome@MCC950 NPs) as the carrier material, with surface modification by the muscle‐homing peptide M12 for targeted delivery to skeletal muscle tissue. Nanoparticle characteristics were assessed using transmission electron microscopy (TEM), dynamic light scattering (DLS) and in vitro drug release assays. The targeting efficiency was evaluated in vivo using fluorescence imaging and in vitro via cellular uptake studies in C2C12 myoblasts. The anti‐inflammatory and anti‐atrophic effects were investigated in an LPS‐induced myotube atrophy model and a cecal ligation and puncture (CLP)‐induced sepsis mouse model. Biocompatibility and systemic safety were assessed through histological analysis and serum biochemical assays.

**Results:**

M12‐Liposome@MCC950 NPs exhibited a uniform spherical morphology, an average diameter of 150 ± 10 nm and a zeta potential of −15.73 ± 6.03 mV, ensuring good colloidal stability. The nanoparticles demonstrated sustained drug release over 14 days. In vivo fluorescence imaging confirmed enhanced skeletal muscle accumulation of M12‐conjugated nanoparticles, with a 3.47‐ to 5.31‐fold increase compared to nontargeted controls. Cellular uptake studies revealed a 2.28‐fold improvement in intracellular delivery efficiency. In vitro, M12‐Liposome@MCC950 NPs significantly inhibited NLRP3 inflammasome activation, reducing caspase‐1 cleavage and IL‐1β/IL‐18 secretion, while also preventing LPS‐induced myotube atrophy. In the CLP‐induced sepsis model, treatment with M12‐Liposome@MCC950 NPs markedly reduced muscle atrophy, improved grip strength and decreased expression of atrophy‐related proteins Atrogin‐1 and MuRF1. Additionally, histological and biochemical assessments confirmed that the nanoparticles did not induce hepatic or renal toxicity, demonstrating excellent biocompatibility.

**Conclusions:**

M12‐Liposome@MCC950 NPs provide a targeted and sustained‐release strategy for delivering MCC950 to skeletal muscle, effectively inhibiting NLRP3 inflammasome activation and alleviating SIM. This approach enhances therapeutic efficacy while mitigating systemic toxicity, highlighting the potential of nanomedicine‐based interventions for treating inflammation‐related myopathies.

## 1 Introduction

Sepsis is a systemic inflammatory response syndrome caused by infection, often leading to multiple organ dysfunction and representing a leading cause of in‐hospital mortality [1]. Sepsis‐induced myopathy (SIM) is a common complication in patients with sepsis, and more than half of those with sepsis and multiple organ failure in intensive care units (ICUs) develop intensive care unit‐acquired weakness (ICUAW) [2, 3]. This myopathy is characterized by muscle weakness, atrophy and fatigue, severely affecting patient recovery and prognosis [4, 5]. ICUAW is one of the critical factors influencing the prognosis of septic patients, as it prolongs ICU stays and increases short‐term mortality [5, 6]. Although supportive strategies such as nutritional supplementation and rehabilitation are currently employed, effective therapeutic interventions are still lacking [7].

The nucleotide‐binding oligomerization domain (NOD)‐like receptor family pyrin domain‐containing 3 (NLRP3) inflammasome acts as a sensor for various signals, including pathogens such as bacteria and viruses, as well as tissue damage‐associated stimuli [8, 9, 10]. In the classical activation pathway, the NLRP3 inflammasome assembles and activates caspase‐1, which subsequently triggers pyroptosis and promotes the maturation and release of pro‐inflammatory cytokines such as interleukin (IL)‐1β and IL‐18, initiating a robust inflammatory response [11, 12, 13]. While moderate activation of the inflammasome facilitates microbial clearance, excessive activation may lead to uncontrolled inflammation and tissue injury [14, 15]. The NLRP3 inflammasome plays a broad role in sepsis by mediating the death of immune cells, including macrophages [16], neutrophils [17] and T lymphocytes [18], as well as contributing to multiple organ dysfunction. It also serves as a key regulator of skeletal muscle metabolism [19, 20, 21]. Emerging evidence suggests that NLRP3 inflammasome activation plays a central role in sepsis‐induced skeletal muscle atrophy. Upon activation, NLRP3 recruits and activates caspase‐1, leading to maturation and secretion of IL‐1β and IL‐18, which trigger local inflammatory responses. These inflammatory mediators activate the UPS, promoting muscle protein degradation. Additionally, NLRP3 activation can induce myofibre pyroptosis and inhibit regenerative signaling, collectively exacerbating muscle atrophy [19, 22, 23].

MCC950 is a highly selective small‐molecule inhibitor of the NLRP3 inflammasome that blocks the conformational change of NLRP3 and its interaction with ASC, thereby suppressing caspase‐1 activation and the maturation and release of IL‐1β and IL‐18 [24]. It has been shown to effectively suppress NLRP3 inflammasome activation and is considered a promising candidate for blocking this pathway [24, 25, 26]. Preclinical studies have demonstrated that MCC950 exhibits significant protective effects in multiple models of sepsis‐related injury. Specifically, studies based on animal models showed that MCC950 alleviates sepsis‐associated multiple organ dysfunction [27] and myocardial injury [26], while both in vitro C2C12 myotube experiments and in vivo CLP‐induced sepsis models confirmed its ability to improve sepsis‐induced skeletal muscle atrophy [24]. Although MCC950 (CP‐456,773) entered clinical trials, development was reportedly terminated due to liver safety concerns. In particular, the total daily dose reached 1200 mg, resulting in plasma Cmax > 100 μM, and the compound exhibits high lipophilicity (ClogP 2.9–4.43), which may contribute to the risk of drug‐induced liver injury (DILI). These factors suggest that high systemic exposure and lipophilicity could limit its clinical application [28, 29].

In recent years, nanotechnology has shown distinct advantages in the targeted delivery of therapeutics for sepsis, not only enhancing drug accumulation in target tissues but also reducing off‐target exposure and associated side effects [30, 31]. In this study, we designed and fabricated a liposome‐based nanoparticle (NP) system, functionalized with the muscle‐homing peptide M12, to achieve targeted delivery of the NLRP3 inhibitor MCC950 (M12‐Liposome@MCC950 NPs). Our in vitro and in vivo experiments demonstrated that the M12‐functionalized NPs significantly enhanced MCC950 enrichment in muscle tissue and cellular uptake efficiency. Furthermore, they effectively inhibited the activation of the NLRP3 signaling pathway and reduced pyroptosis and atrophy of muscle cells. The nanoplatform not only exhibited favourable skeletal muscle‐targeting capability and extended duration of action but also improved therapeutic efficacy while minimizing systemic toxicity.

### 1.1 Materials

MCC950 (Synonyms: CP‐456773; CRID3) was purchased from MCE (Monmouth Junction, NJ, USA). 1,2‐dipalmitoyl‐sn‐glycero‐3‐phosphocholine (DPPC), cholesterol, 1,2‐distearoyl‐sn‐glycero‐3‐phosphoethanolamine‐N‐[methoxy(polyethylene glycol)‐2000] (Ammonium Salt) (DSPE‐PEG2k) and 1,2‐distearoyl‐sn‐glycero‐3‐phosphoethanolamine‐N‐[succinimidyl(polyethylene glycol)] (DSPE‐PEG2k‐NHS). M12 peptide (RRQPPRSISSHP, MW: 1417.6 Da) was purchased from Thermo Fisher Scientific (Waltham, MA, USA). HRP‐conjugated secondary antibodies, including anti‐rabbit IgG (Catalogue #7074S) and anti‐mouse IgG (Catalogue #7076S), were purchased from Cell Signaling Technology (Danvers, MA, USA).

All organic solvents used in the experiments were purchased from Thermo Fisher Scientific (Waltham, MA, USA). All other reagents were obtained from Sigma‐Aldrich (St. Louis, MO, USA).

### 1.2 Preparation/Functionalization and Characterization of Liposome

To obtain MCC950‐loaded liposome, the mixture of 1,2‐dipalmitoyl‐sn‐glycero‐3‐phosphocholine (DPPC), cholesterol and 1,2‐distearoyl‐sn‐glycero‐3‐phosphoethanolamine‐N‐[succinimidyl(polyethylene glycol)] (DSPE‐PEG2k‐NHS) at a molar ratio of 15:7.5:2 was dissolved in chloroform and then dried under a rotary evaporator. Afterward, the dried lipid film was hydrated with MCC950 (1 mg/mL), followed by ultrasound for 20 min. The resulting Liposome@MCC950 was condensed with an Amico filter device with a molecular weight cut‐off of 30 kDa (Millipore) for further use.

#### 1.2.1 Functionalization: Muscle‐Homing Peptide Conjugation

To achieve muscle‐targeted delivery, the muscle‐homing peptide M12 (RRQPPRSISSHP) was conjugated to the Liposome. Briefly, 1 mg of peptide was added to the liposome (2 mg/mL), and the pH was adjusted to 8.3 using 1‐M sodium bicarbonate buffer. The mixture was stirred overnight at 4°C. Unbound peptides were removed by centrifugation and triple washing.

#### 1.2.2 Characterization

Particle size, polydispersity index (PDI) and zeta potential were measured using dynamic light scattering (DLS; Malvern Nano ZS, Malvern Instruments, Worcestershire, UK). Morphology and size were observed by transmission electron microscopy (TEM; Tecnai G2 20, FEI Company, Hillsboro, USA), with samples stained using 1% phosphotungstic acid. The MCC950 content was quantified using a microplate reader (Synergy H1, Biotek Instruments, Winooski, USA) at a wavelength of 302 nm.

#### 1.2.3 In Vitro Drug Release

The in vitro release behaviour of MCC950 was evaluated by dialysis. A specific amount of MCC950‐loaded liposome was suspended in 1‐mL phosphate‐buffered saline (PBS, pH 7.4 or 6.5, as per experimental design), transferred into dialysis bags (molecular weight cut‐off 12–14 kDa) and immersed in 50‐mL release medium (PBS containing 0.5% Tween‐80 to maintain sink conditions). The set‐up was incubated at 37°C under gentle agitation (100 rpm). At predetermined time points, 1 mL of the release medium was withdrawn and replaced with an equal volume of prewarmed fresh medium. The concentration of MCC950 was determined by high‐performance liquid chromatography (HPLC), and cumulative release (%) was calculated as follows:

$$\begin{array}{c} \begin{array}{c} \begin{array}{c} \text{Cumulative release}\;\left(\%\right)=\left(\mathrm{Ct}\times V+\sum \text{Cprev}\times V^{\prime }\right)/\text{Total drug content}\times 100 \end{array} \end{array} \end{array}$$

where Ct is the drug concentration at each time point, V is the total volume of the release medium, V′ is the sample volume and ∑Cprev is the sum of concentrations at previous time points. All experiments were performed in triplicate, and data were expressed as mean ± standard deviation (SD).

### 1.3 C2C12 Cell Culture and Treatment

C2C12 murine myoblasts were obtained from Wuhan Procell Life Science & Technology Co. Ltd. Cells were cultured in high‐glucose Dulbecco's Modified Eagle Medium (DMEM; Gibco, USA) supplemented with 10% foetal bovine serum (FBS; Gibco, USA) and 1% penicillin–streptomycin (100‐U/mL penicillin and 100‐μg/mL streptomycin; Gibco, USA), maintained at 37°C in a humidified incubator with 5% CO_2_. Cells were subcultured at a ratio of 1:3, with medium replaced regularly. Experiments were conducted when cells reached 70%–80% confluency.

### 1.4 Cellular Uptake and Intracellular Localization

C2C12 cells were seeded at 5 × 10^4^ cells/well in 12‐well plates and incubated for 24 h. After PBS washing, cells were incubated with DMEM containing Nile red‐labeled NPs (0.1 mg/mL) for 1 h. Nuclei were stained with Hoechst 33342 (1 μg/mL in PBS) for 10 min at 37°C, followed by PBS washes to remove excess particles and dead cells. Fluorescent signals were visualized by fluorescence microscopy. For intracellular localization, organelle‐specific dyes (e.g., MitoTracker, LysoTracker or Calreticulin antibody) were used, followed by confocal microscopy. Quantification of intracellular uptake was performed using corrected total cell fluorescence (CTCF) with ImageJ software. All experiments were repeated independently three times.

### 1.5 In Vivo Biodistribution of NPs

DiI‐labeled Liposomes (DiI‐Liposome@MCC950 or DiI‐M12‐Liposome@MCC950) were intravenously injected into mice via the tail vein at a dose of 10 mg/kg. After 6 h, mice were anaesthetised by intraperitoneal injection of ketamine, and hair was removed from both hind limbs. Background fluorescence was adjusted using untreated mice. Whole‐body fluorescence imaging was conducted using an Ami imaging system (Spectral Instruments Imaging, Tucson, USA). After imaging, mice were euthanized, and major organs (liver, kidney, spleen, heart, quadriceps, gastrocnemius and diaphragm) were collected for ex vivo imaging. Fluorescence intensity ratios between muscle tissues and liver were calculated using Living Image software.

### 1.6 Cell Viability Assay (CCK‐8)

C2C12 myotubes were seeded at 1 × 10^4^ cells/well in 96‐well plates. After treatment, 100 μL of CCK‐8 solution (10 μg/mL; MCE, China) was added to each well and incubated at 37°C for 2 h. Absorbance was measured at 450 nm using a microplate reader (Multiskan FC, Thermo Fisher, USA), and relative viability was calculated according to the manufacturer's protocol.

### 1.7 CLP‐Induced Sepsis Animal Model

All animal procedures were approved by the Animal Ethics Committee of Tongji Hospital, Huazhong University of Science and Technology (Approval No.: T1‐202505022). Twelve‐week‐old male Balb/c mice (22–26 g) were obtained from Beijing Vital River Laboratory Animal Technology Co. Ltd. and housed under SPF conditions with a 12‐h light/dark cycle and ad libitum access to food and water. Sepsis was induced by cecal ligation and puncture (CLP) performed by an experienced surgeon [32, 33]. Mice were euthanized at predetermined time points, and tissues were collected for further analysis.

### 1.8 Grip Strength Test

Forelimb grip strength was measured using a grip strength metre. Mice grasped a T‐bar with their forelimbs, and the experimenter gently pulled the tail along a horizontal axis. The maximum force exerted before release was recorded. Data were analysed according to the instrument software instructions.

### 1.9 Protein Extraction and Western Blot

C2C12 myotubes or gastrocnemius tissues were lysed in precooled RIPA buffer. Total protein was collected by centrifugation at 12 000 × *g* for 15 min at 4°C. A total of 25 μg of protein per sample was loaded for SDS–PAGE, separated on 8%–15% gels and transferred onto PVDF membranes. After blocking with 5% nonfat milk for 1 h, membranes were incubated overnight at 4°C with primary antibodies (1:1000, Abclonal, China; Abcam, UK; Abmart, China), followed by HRP‐conjugated secondary antibodies (1:10 000, Seville Biotechnology, China) for 1 h at room temperature. Bands were visualized using Clarity Western ECL substrate and imaged with a ChemiDoc XRS+ system (Bio‐Rad, USA). Band intensity was quantified using Image Lab 6.0 software. Detailed antibody information is provided in Table S1.

### 1.10 Haematoxylin and Eosin (H&E) Staining

Fresh gastrocnemius tissues were fixed in 4% paraformaldehyde overnight, embedded in paraffin and sectioned at 4‐μm thickness for H&E staining. H&E‐stained sections were imaged at 20× magnification using a light microscope (Zeiss Axio Scope A1, Germany) equipped with a digital camera. For each muscle sample, five random images were captured. The cross‐sectional area (CSA) of muscle fibres was analysed using ImageJ software. The mean CSA was determined by measuring 50 adjacent myofibres per image, totaling 250 fibres per sample and 1250 fibres per experimental group.

### 1.11 Statistical Analysis

All data were presented as mean ± SD. Statistical analysis was performed using GraphPad Prism 8.0 (GraphPad Software, USA). Comparisons between two groups were conducted using Student's *t*‐test.

## 2 Results

### 2.1 Particle Size, Morphology and Surface Characteristics of the NPs

**FIGURE 1:**
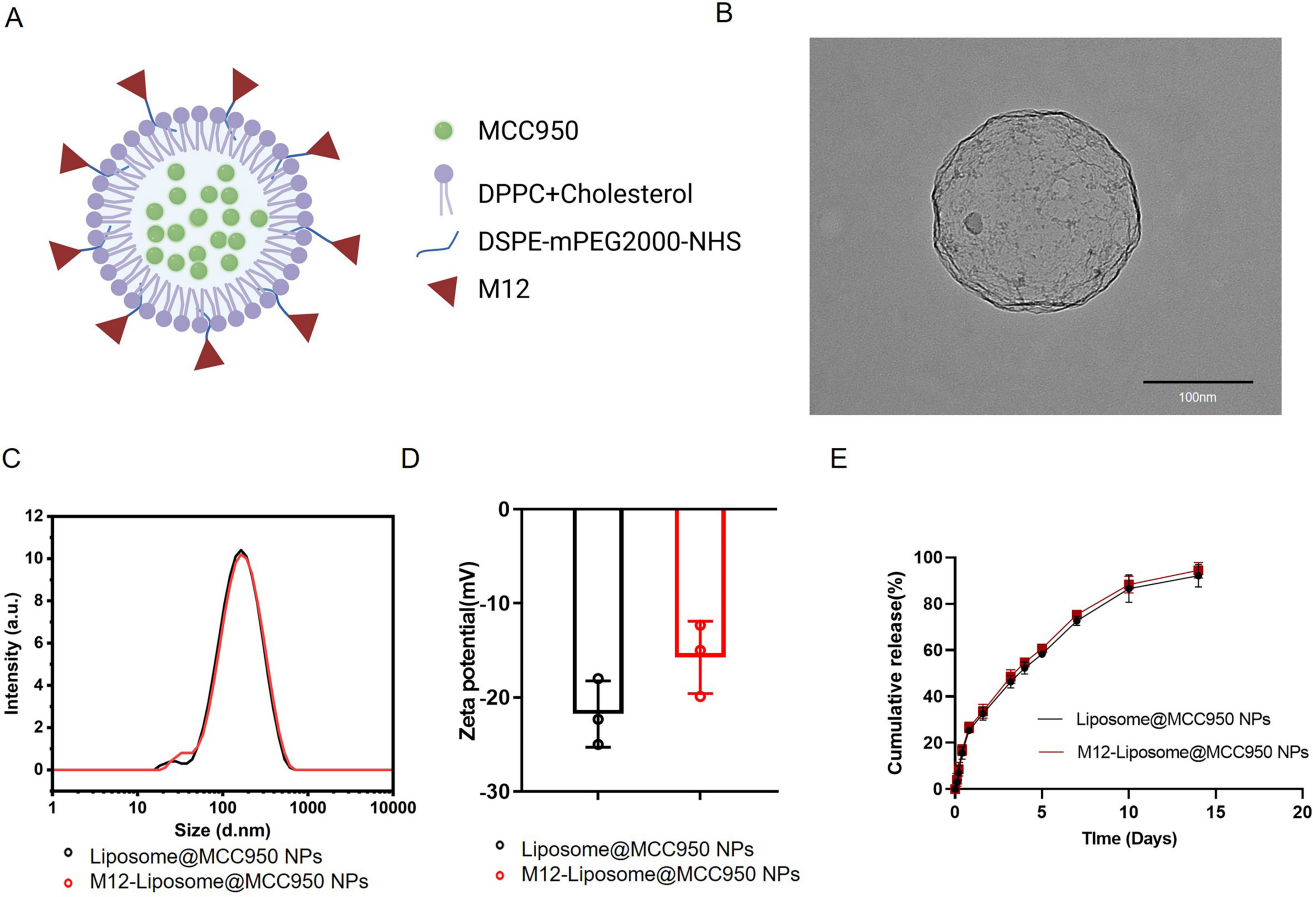
Size, morphology and surface characteristics of nanoparticles. (A) Schematic diagram of MCC950‐loaded M12‐Liposome nanoparticles (M12‐Liposome@MCC950 NPs). (B) Representative transmission electron microscopy (TEM) image of M12‐Liposome@MCC950 NPs. (C) Size distribution of M12‐Liposome@MCC950 NPs measured by dynamic light scattering (DLS). (D) Zeta potential analysis. (E) In vitro release profiles of MCC950 from liposome and M12‐Liposome nanoparticles.

MCC950 was encapsulated into liposomes using the solvent evaporation method, achieving an encapsulation efficiency of 65.3%. A schematic diagram of the MCC950‐loaded M12‐Liposome is shown in Figure 1A. The muscle‐targeting peptide M12 was covalently conjugated to liposomes via N‐hydroxysuccinimide (NHS) ester reactions, where the N‐terminal α‐amino group of M12 was conjugated to DSPE‐PEG2k‐NHS. M12 has been proven to possess a high affinity for skeletal and cardiac muscle, facilitating effective delivery of the loaded drug to target tissues following systemic administration.

TEM revealed spherical morphology of the liposomes (Figure 1B). After M12 conjugation, the density distribution of the NPs appeared uneven, indicating the successful surface functionalization of the liposome with M12. DLS analysis demonstrated that the average size of the M12‐Liposome@MCC950 NPs was 150 ± 10 nm with a narrow size distribution and a PDI of 0.067 ± 0.018 (Figure 1C). Additionally, the zeta potential was −15.73 ± 6.03 mV, suggesting good colloidal stability and dispersion ability (Figure 1D).

In vitro drug release profiles in PBS (pH 7.4, 37°C) showed sustained release of MCC950 over 14 days (Figure 1E). More than 50% of the drug was released within the first 3 days, followed by a gradual release thereafter. M12 conjugation did not significantly alter the release kinetics of MCC950, indicating that peptide modification did not affect the drug release behaviour.

### 2.2 Cellular Uptake Efficiency of M12‐Liposome@MCC950 NPs in Skeletal Muscle and Myoblasts

**FIGURE 2:**
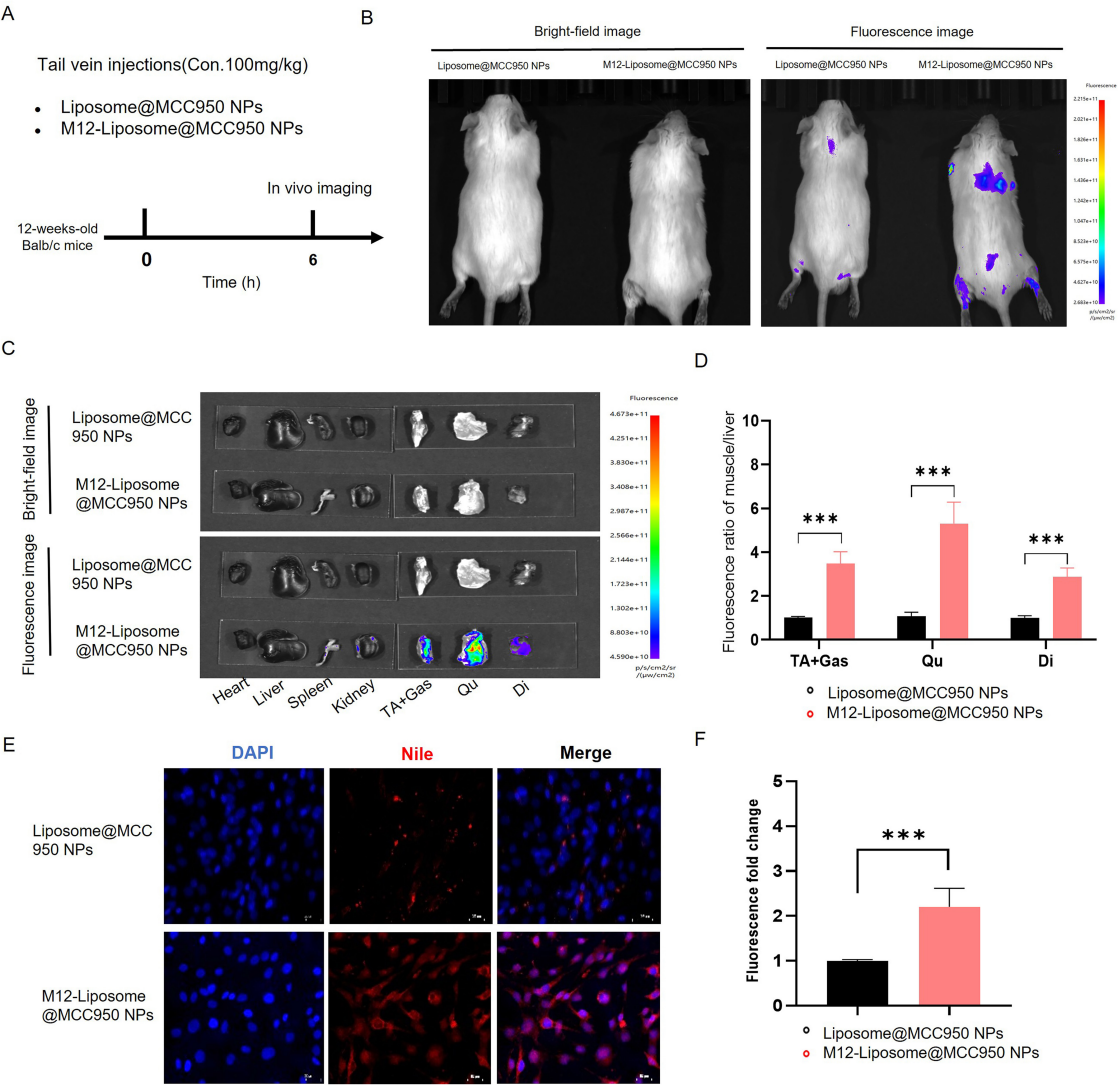
Muscle‐targeting capacity of M12‐modified nanoparticles following systemic administration. (A) Experimental design and timeline of nanoparticle injection in Balb/c mice. (B) Whole‐body fluorescence imaging at 6 h post‐injection using DiI‐Liposome@MCC950 or DiI‐M12‐Liposome@MCC950. (C) Biodistribution of fluorescently labeled NPs in various tissues, including heart, liver, kidney, spleen, quadriceps (Qu), tibialis anterior (TA), gastrocnemius (Gas) and diaphragm (Di). (D) Quantitative analysis of muscle‐to‐liver fluorescence intensity ratios. (E) Representative fluorescence microscopy images of C2C12 myotubes treated with Nile red‐labeled liposome NPs or M12‐Liposome NPs (0.1 mg/mL, 1 h). (F) Quantitative analysis of nanoparticle uptake in C2C12 myotubes. NS: no significant difference; ***p < 0.001. Data are presented as mean ± SEM (*n* = 4).

To evaluate the in vivo targeting capability of M12‐Liposome@MCC950 NPs, mice were intravenously injected with DiI‐Liposome@MCC950 or DiI‐M12‐Liposome@MCC950, and whole‐body fluorescence imaging was performed (Figure 2A). Six hours post‐injection, strong fluorescence signals were observed in the limbs of mice injected with M12‐conjugated NPs compared to nontargeted controls (Figure 2B). Ex vivo imaging of dissected tissues (heart, liver, kidneys, spleen and skeletal muscles) revealed that DiI‐M12‐Liposome@MCC950 NPs predominantly accumulated in skeletal muscles such as the quadriceps, tibialis anterior, gastrocnemius and diaphragm (Figure 2C). Quantitative analysis normalized to liver fluorescence intensity showed 3.47‐, 5.31‐ and 2.88‐fold higher accumulation in the tibialis anterior + gastrocnemius, quadriceps and diaphragm, respectively, with statistical significance (*p* ≤ 0.001; Figure 2D). To assess the active targeting ability of M12‐Liposome@MCC950 to muscle cells, C2C12 cells were incubated with either Liposome@MCC950 NPs or M12‐Liposome@MCC950 NPs, with Nile Red used for fluorescent labeling. After 4 h, red fluorescence was observed mainly in the cytoplasm and perinuclear region, indicating efficient cellular uptake (Figure 2E). Notably, M12‐conjugated NPs showed stronger intracellular fluorescence. Quantitative analysis confirmed a 2.28‐fold increase in uptake efficiency for M12‐Liposome@MCC950 NPs (Figure 2F).

### 2.3 In Vitro Biocompatibility and Anti‐Atrophy Effects of M12‐Liposome@MCC950 NPs

**FIGURE 3:**
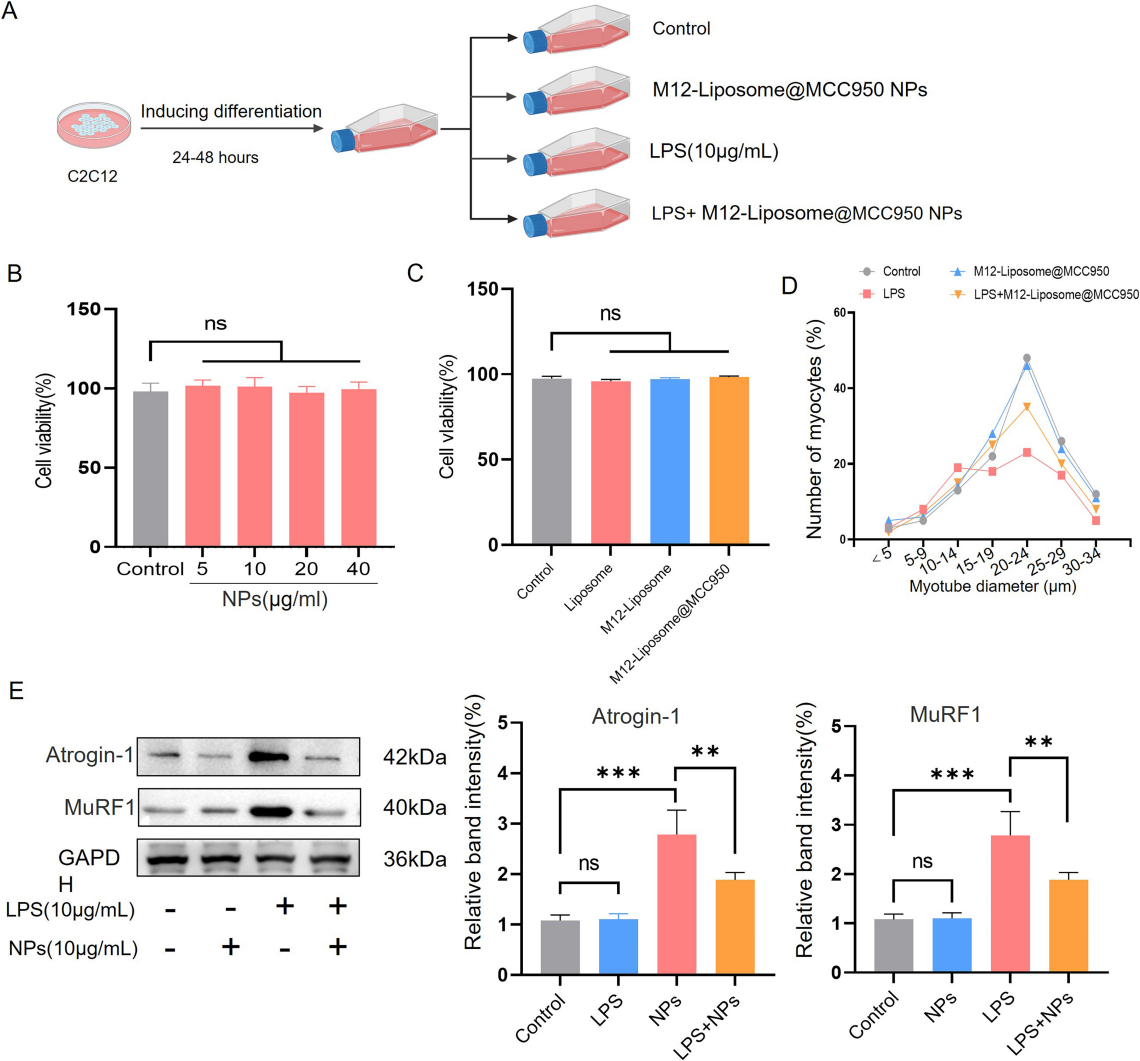
Effects of M12‐Liposome@MCC950 NPs on myotube viability and atrophy. (A) Schematic of experimental design. (B) Cell viability of C2C12 myotubes treated with different concentrations of M12‐Liposome@MCC950 NPs (5, 10, 20 and 40 μg/mL) for 48 h. (C) Cell viability of C2C12 myotubes after treatment with liposome, M12‐Liposome or M12‐Liposome@MCC950 NPs at a working concentration of 10 μg/mL for 48 h, compared with the untreated control. (D) Effects on myotube number after 48 h co‐culture with various formulations. (E) Western blot analysis of muscle atrophy‐related protein expression in different groups and quantification of Western blot results. NS: no significant difference; **p < 0.01, ***p < 0.001. Data are presented as mean ± SEM (*n* = 4).

To assess the biocompatibility of M12‐Liposome@MCC950 NPs, CCK‐8 assays were conducted in C2C12 cells treated with various concentrations of NPs (Figure 3A). As shown in Figure 3B, cell viability remained unaffected at concentrations ranging from 5 to 40 μg/mL (*p* > 0.05), indicating good biocompatibility. Comparison among different groups (control, liposome, M12‐liposome and M12‐Liposome@MCC950) revealed no significant differences in cell viability (*p* > 0.05; Figure 3C), further supporting the low cytotoxicity of the delivery system.

Atrogin‐1 and MuRF1 are muscle‐specific E3 ubiquitin ligases that function as upstream regulators of the ubiquitin–proteasome system (UPS). Their upregulation activates the UPS pathway, leading to accelerated skeletal muscle protein degradation and atrophy [34]. An LPS‐induced C2C12 atrophy model was established to evaluate the anti‐atrophy effect of M12‐Liposome@MCC950 NPs. As shown in Figure 3D, treatment with LPS (10 μg/mL) significantly reduced C2C12 myotube diameter, indicating the induction of atrophy, whereas subsequent treatment with M12‐Liposome@MCC950 NPs effectively restored myotube size. Western blot analysis further confirmed these findings (Figure 3E). LPS significantly upregulated Atrogin‐1 and MuRF1 expression, while M12‐Liposome@MCC950 NPs markedly suppressed these atrophy‐related proteins (Figure 3E), suggesting effective inhibition of LPS‐induced muscle protein degradation.

### 2.4 M12‐Liposome@MCC950 NPs Inhibit LPS‐Induced Activation of the NLRP3 Inflammasome in C2C12 Myotubes

**FIGURE 4:**
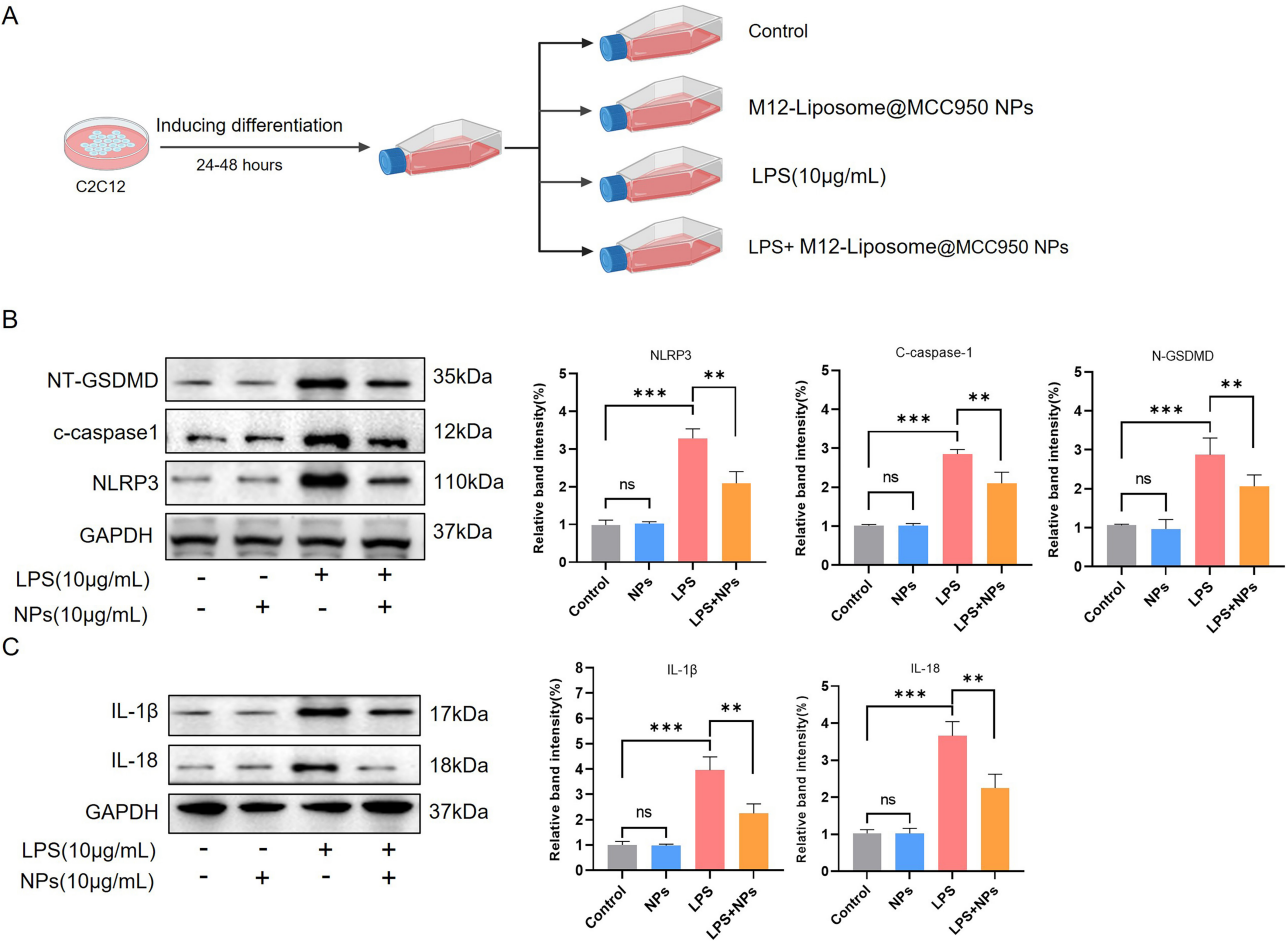
M12‐Liposome@MCC950 NPs alleviate LPS‐induced NLRP3 inflammasome‐mediated inflammation in C2C12 myotubes. (A) Schematic illustration of the experimental workflow. (B) Western blot analysis of NLRP3, cleaved caspase‐1 (c‐caspase‐1) and NT‐GSDMD in C2C12 myotubes treated with LPS (10 μg/mL) with or without nanoparticles(10 μg/mL). (C) Expression levels of inflammatory cytokines IL‐1β and IL‐18 under different treatments. M12‐Liposome@MCC950 NPs (10 μg/mL) markedly attenuated LPS‐induced inflammasome activation and cytokine production. NS: no significant difference; **p < 0.01, ***p < 0.001. Data are presented as mean ± SEM (*n* = 4).

The NLRP3 inflammasome is a key mediator of inflammation and pyroptosis in sepsis‐induced skeletal muscle injury. To evaluate whether M12‐Liposome@MCC950 NPs suppress LPS‐induced inflammasome activation, differentiated C2C12 myotubes were treated with LPS (10 μg/mL) in the presence or absence of different NP formulations (Figure 4A).

As shown in Figure 4B, LPS stimulation markedly increased the expression of NLRP3, cleaved caspase‐1 (c‐caspase‐1) and N‐terminal gasdermin D (NT‐GSDMD), demonstrating activation of the inflammasome and induction of pyroptosis. Treatment with M12‐Liposome@MCC950 NPs (10 μg/mL) significantly reduced the levels of these pyroptosis‐related proteins, indicating effective suppression of inflammasome activation. In addition, LPS exposure elevated the production of inflammatory cytokines IL‐1β and IL‐18. As shown in Figure 4C, M12‐Liposome@MCC950 NPs (10 μg/mL) markedly attenuated LPS‐induced increases in IL‐1β and IL‐18, further confirming their anti‐inflammatory effects.

### 2.5 M12‐Liposome@MCC950 NPs Inhibit LPS‐Induced Activation of the NLRP3 Inflammasome In Vivo

**FIGURE 5:**
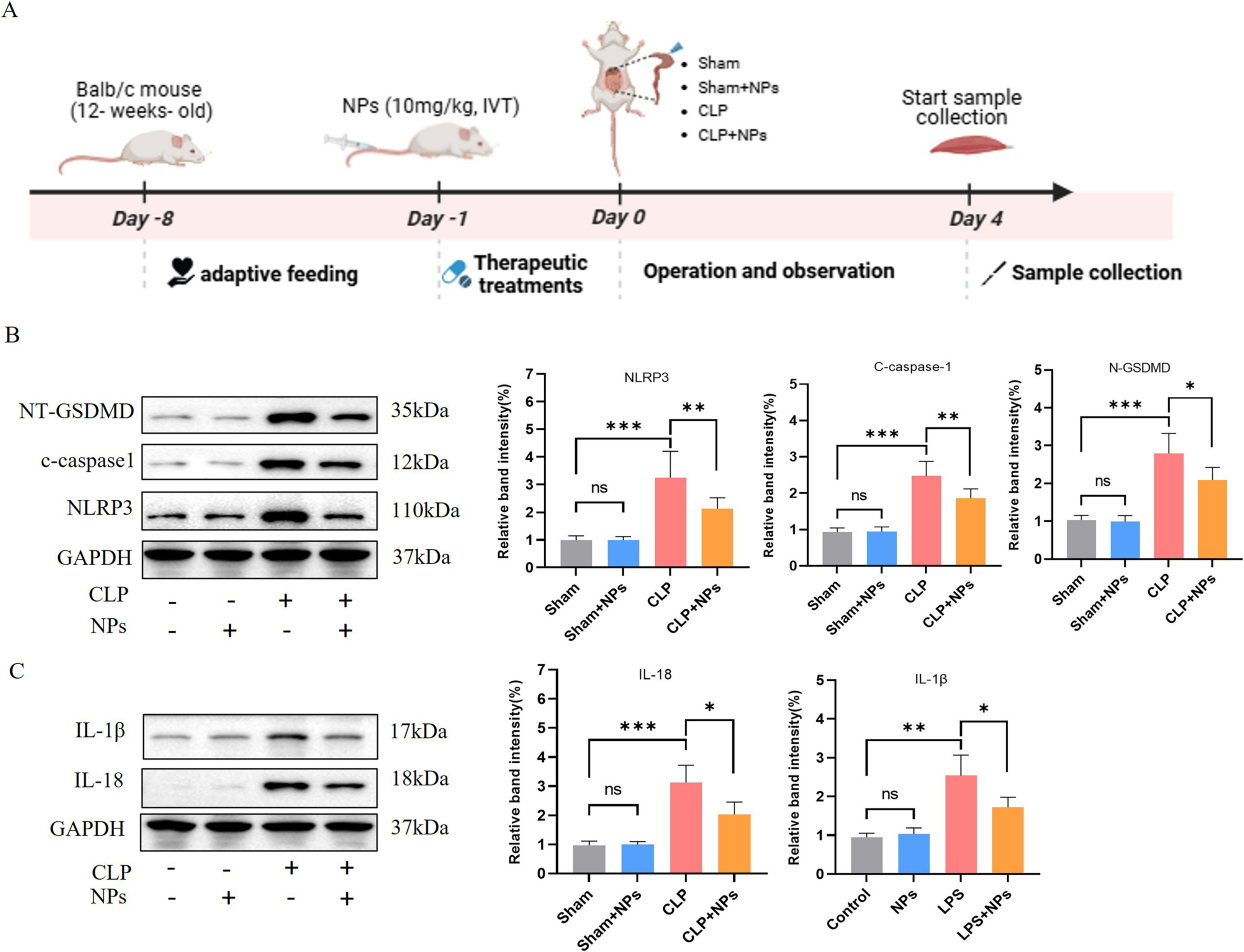
M12‐Liposome@MCC950 NPs inhibit NLRP3 inflammasome activation in a septic skeletal muscle atrophy model. (A) Schematic of experimental design. (B) Muscle tissues were collected on the 4th day after the intervention. Western blot analysis of NLRP3, cleaved caspase‐1 and NT‐GSDMD in muscle tissues. (C) Western blot analysis of IL‐1β and IL‐18 expression. NS: no significant difference; **p < 0.01, ***p < 0.001. Data are presented as mean ± SEM (*n* = 4).

To further validate the in vivo anti‐inflammatory efficacy of the MCC950 delivery system, a CLP‐induced sepsis mouse model was established, and treatment was administered using M12‐Liposome@MCC950 NPs (Figure 5A).

Western blot analysis showed that CLP significantly upregulated NLRP3, cleaved caspase‐1 and NT‐GSDMD in skeletal muscle tissues, confirming inflammasome activation and pyroptosis (Figure 5B). M12‐Liposome@MCC950 NPs treatment markedly decreased the expression of these proteins. Similarly, CLP increased IL‐1β and IL‐18 levels in muscle tissues, which were significantly reduced upon treatment (Figure 5C). These results indicate that M12‐Liposome@MCC950 NPs enhance the bioavailability and therapeutic effect of MCC950 by targeting and suppressing NLRP3 inflammasome‐mediated inflammatory responses.

### 2.6 M12‐Liposome@MCC950 NPs Improve Weight and Skeletal Muscle Status in Septic Mice

**FIGURE 6:**
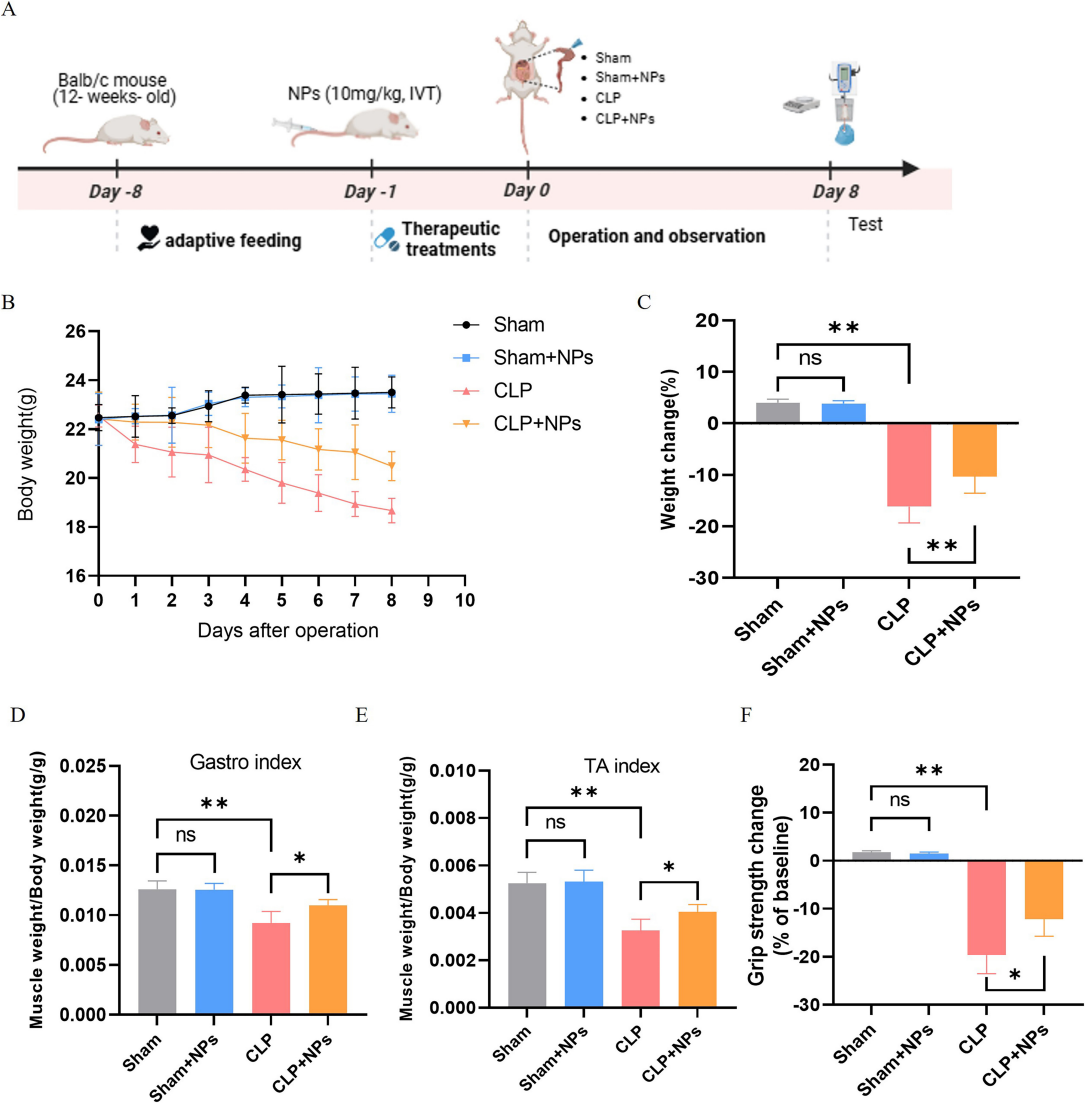
M12‐Liposome@MCC950 NPs protect against CLP‐induced skeletal muscle weakness. (A) Schematic of the CLP‐induced sepsis model, nanoparticle treatment schedule and sample collection. After the intervention, body weight changes were observed over an 8‐day period (B), and forelimb grip strength test was performed for muscle function evaluation (C). The muscle index (muscle weight/body weight) was observed for the gastrocnemius (D) and tibialis anterior (E). (F) Grip strength testing. NS: no significant difference; *p < 0.05, **p < 0.01, ***p < 0.001. Data are presented as mean ± SEM (*n* = 5).

To evaluate the effect of M12‐Liposome@MCC950 NPs on sepsis‐induced skeletal muscle atrophy, multiple physiological and muscle‐related indicators were assessed (Figure 6A–E). Body weight monitoring revealed that the CLP group exhibited continuous postoperative weight loss, whereas treatment with M12‐Liposome@MCC950 NPs significantly alleviated this trend (Figure 6B,C). Furthermore, measurements of the gastrocnemius (GA) and tibialis anterior (TA) muscle mass further confirmed the protective effect of these NPs against muscle atrophy (Figure 6D,E). Grip strength testing demonstrated that, compared to the CLP group, mice treated with M12‐Liposome@MCC950 NPs exhibited significantly improved muscle strength (Figure 6F), suggesting the potential therapeutic value of this nanomedicine in alleviating sepsis‐associated muscle dysfunction.

### 2.7 The Protective Effect of M12‐Liposome@MCC950 NPs on CLP‐Induced Skeletal Muscle Atrophy

**FIGURE 7:**
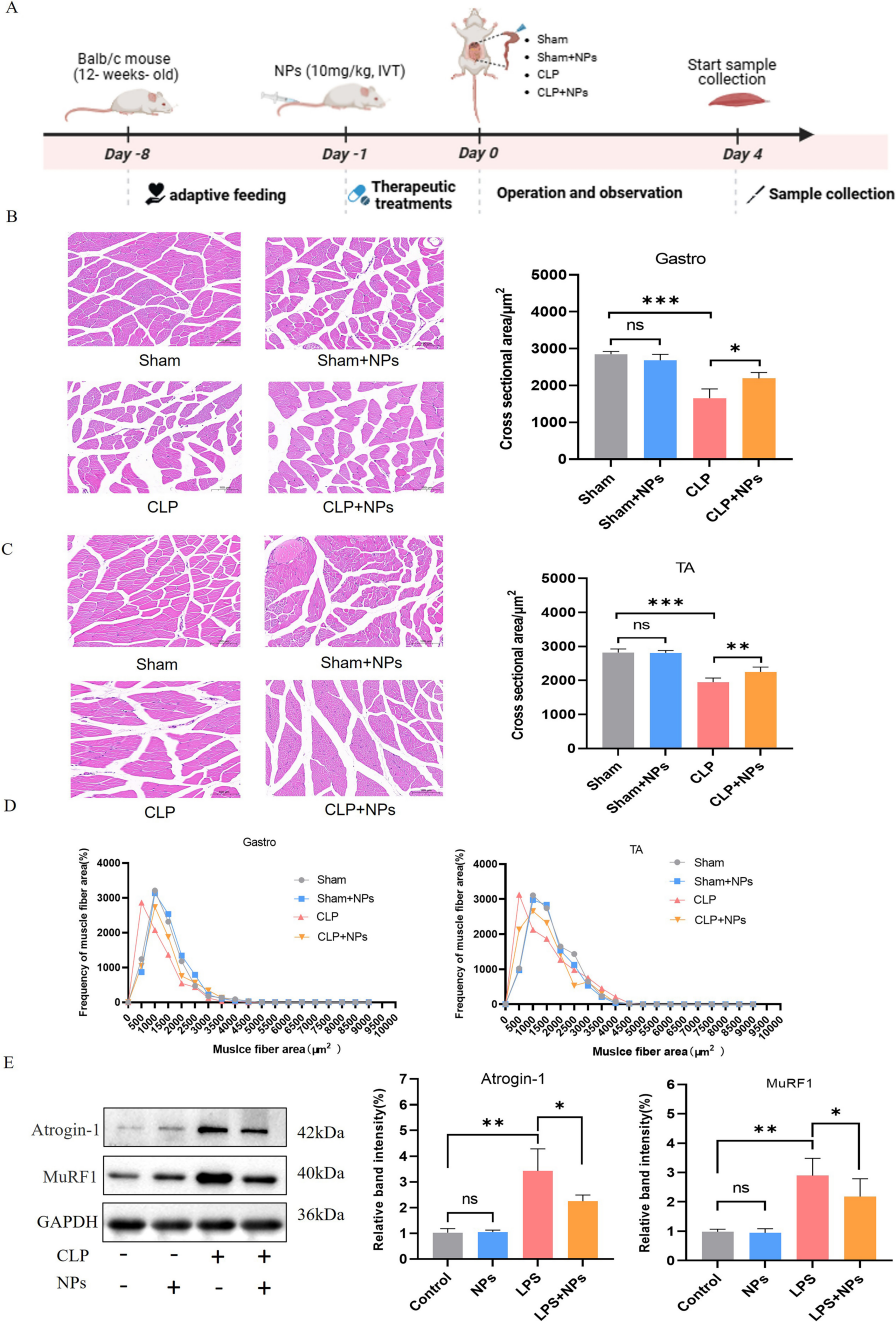
M12‐Liposome@MCC950 NPs alleviate CLP‐induced skeletal muscle atrophy. Schematic of the CLP‐induced sepsis model, nanoparticle treatment schedule and sample collection. H&E staining of gastrocnemius and tibialis anterior muscles (×200 magnification). Frequency distribution of muscle fibre cross‐sectional area (CSA). Western blot analysis of muscle atrophy markers. NS: no significant difference; *p < 0.05, **p < 0.01, ***p < 0.001. Data are presented as mean ± SEM (*n* = 5).

To investigate the protective effect of M12‐Liposome@MCC950 NPs. The experimental timeline is shown in Figure 7A, where BALB/c mice underwent adaptive feeding before CLP surgery and received a single dose of M12‐Liposome@MCC950 NPs (10 mg/kg, IVT) 1 day before surgery. Samples were collected on Day 4 post‐surgery for analysis. H&E staining results (Figure 7B,C) showed a significant reduction in the CSA of the gastrocnemius (Gastro) and tibialis anterior (TA) muscles in the CLP group, whereas treatment with M12‐Liposome@MCC950 NPs alleviated muscle fibre atrophy. Statistical analysis indicated that compared with the Sham group, the muscle fibre CSA in the CLP group was significantly reduced, whereas it was notably increased in the CLP + NPs group compared with the CLP group. These findings suggest that M12‐Liposome@MCC950 NPs exert a protective effect against CLP‐induced skeletal muscle atrophy. Muscle fibre area distribution analysis (Figure 7D) further supported these results, showing that muscle fibres in the CLP group were primarily concentrated in a smaller size range. However, after NPs treatment, the muscle fibre distribution curve shifted to the right, indicating a reduction in muscle fibre atrophy. Additionally, western blot analysis (Figure 7E) demonstrated that the expression levels of muscle atrophy‐related proteins Atrogin‐1 and MuRF1 were significantly upregulated in the CLP group. However, M12‐Liposome@MCC950 NPs treatment reduced the expression levels of these proteins, suggesting that the NPs might mitigate CLP‐induced skeletal muscle degradation by inhibiting the ubiquitin–proteasome pathway. The results indicate that M12‐Liposome@MCC950 NPs effectively alleviate CLP‐induced skeletal muscle atrophy, potentially by suppressing the expression of muscle degradation‐related proteins.

### 2.8 Safety Evaluation of M12‐Liposome@MCC950 NPs in Mice

MCC950, a known NLRP3 inflammasome inhibitor, has entered phase II clinical trials for rheumatoid arthritis. However, studies have reported its potential hepatotoxicity, as evidenced by elevated serum liver enzyme levels. Therefore, we further evaluated the biosafety of M12.

**FIGURE 8:**
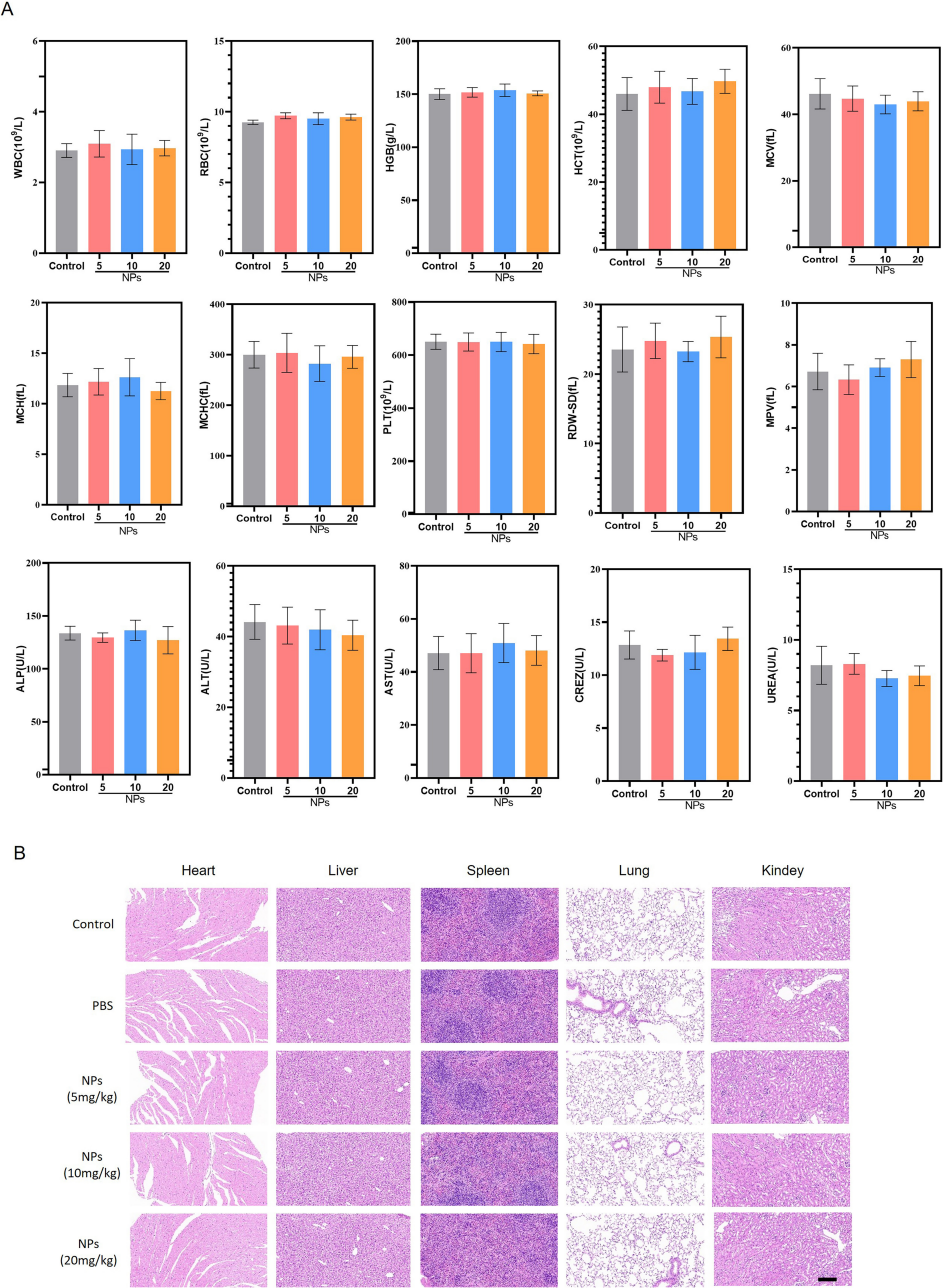
(A) The effects of M12‐Liposome@MCC950 NPs on the haematological system and biochemical parameters. Blood biochemical and routine blood test index levels. WBC: white blood cell, RBC: red blood cell, HGB: haemoglobin, HCT: haematocrit, MCV: mean corpuscular volume, MCH: mean corpuscular haemoglobin, MCHC: mean corpuscular haemoglobin concentration, PLT: platelet count, RDW‐SD: red cell distribution width‐standard deviation, MPV: mean platelet volume, ALP: alkaline phosphatase, ALT: alanine aminotransferase, AST: aspartate aminotransferase, CRE: creatinine. (B) The pathological effects of M12‐Liposome@MCC950 NPs on visceral organs. H&E staining of major organ tissues (heart, liver, spleen, lungs and kidneys).

The haematological parameters, including white blood cell (WBC), red blood cell (RBC), haemoglobin (HGB), haematocrit (HCT), mean corpuscular volume (MCV), mean corpuscular haemoglobin (MCH), mean corpuscular haemoglobin concentration (MCHC), platelet count (PLT), red cell distribution width‐standard deviation (RDW‐SD) and mean platelet volume (MPV), exhibited no significant differences among the control, PBS and NP treatment groups at different doses (5, 10 and 20 mg/kg). Similarly, the biochemical markers related to liver and kidney function, such as alkaline phosphatase (ALP), alanine aminotransferase (ALT), aspartate aminotransferase (AST), creatinine (CRE) and urea, showed no substantial alterations across the groups, suggesting that the administration of M12‐Liposome@MCC950 NPs does not induce haematological or biochemical toxicity (Figure 8A).

Histological examination of major organs, including the heart, liver, spleen, lung and kidney, revealed no significant pathological changes in any of the NP‐treated groups compared to the control and PBS groups. The tissue architecture remained intact, with no visible signs of inflammation, necrosis or structural damage, further supporting the biocompatibility and safety of the NPs at the tested doses (Figure 8B). The results indicate that M12‐Liposome@MCC950 NPs exhibit excellent biocompatibility and do not cause adverse haematological, biochemical or histopathological effects in mice.

## 3 Discussion

In recent years, MCC950, a specific inhibitor of the NLRP3 inflammasome, has demonstrated promising therapeutic effects in various inflammation‐related disease models [24, 35, 36] and is considered a potential agent for the treatment of SIM [26, 27, 37]. However, its clinical translation has been hindered by hepatotoxicity associated with systemic administration [28]. To address this limitation, we developed a functionalized NP delivery system—M12‐Liposome@MCC950 NPs to specifically deliver MCC950 to skeletal muscle tissue, thereby alleviating SIM.

Our results demonstrated that the nanocarrier significantly enhanced the accumulation of MCC950 in skeletal muscle both in vitro and in vivo, improved cellular uptake efficiency and effectively inhibited NLRP3‐mediated inflammation and pyroptosis, thereby attenuating sepsis‐induced muscle atrophy and functional impairment. Compared with free MCC950, the use of liposome NPs functionalized with M12 peptide improved targeting specificity, reduced off‐target distribution and systemic toxicity and enhanced the therapeutic efficacy and translational potential of MCC950.

The M12‐Liposome@MCC950 NPs were successfully prepared via the emulsion‐solvent evaporation method and characterized by TEM and DLS. The NPs exhibited a spherical morphology with a uniform size (approximately 150 ± 10 nm) and a moderately negative surface charge (zeta potential: −15.73 ± 6.03 mV), which contributes to good dispersibility and stability. The conjugation of M12 peptide did not significantly alter the release profile of MCC950 from the liposome carrier, which maintained a sustained release over 14 days, helping to maintain effective drug concentrations at the target site and improving therapeutic outcomes.

Efficient cellular uptake is essential for effective drug delivery. In line with previous studies [38], we employed the muscle‐targeting peptide M12 as a functional ligand to enhance skeletal muscle‐specific delivery. In vivo biodistribution analysis confirmed that M12 modification significantly increased MCC950 accumulation in skeletal muscle tissue. Fluorescence imaging showed that M12‐Liposome@MCC950 NPs exhibited a 2.88–5.31‐fold stronger signal in skeletal muscles (quadriceps, tibialis anterior, gastrocnemius and diaphragm) compared to unmodified NPs, indicating the effective targeting capability of the M12 peptide. Furthermore, cellular experiments showed a 2.28‐fold increase in the uptake of M12‐modified NPs in C2C12 cells, demonstrating the efficiency of M12‐mediated targeted delivery and its contribution to enhanced therapeutic efficacy. Importantly, although IV‐administered M12‐modified NPs also exhibited a detectable fluorescence signal in the heart, this cardiac enrichment likely reflects transient perfusion‐associated accumulation during systemic circulation rather than stable tissue uptake. Our ex vivo quantification and histologic analyses detected minimal long‐term cardiac retention and no cardiac toxicity, demonstrating that the fluorescence observed in the heart is not indicative of functional delivery.

We further systematically evaluated the therapeutic potential of the NPs in relieving SIM and verified the pivotal role of NLRP3 inflammasome activation in septic skeletal muscle injury. The M12‐Liposome@MCC950 NPs exhibited excellent biocompatibility and effectively inhibited LPS‐induced myotube atrophy in C2C12 cells. CCK‐8 assays indicated no obvious cytotoxicity in the concentration range of 5–40 μg/mL. Western blot analysis revealed that M12‐Liposome@MCC950 NPs significantly downregulated the expression of muscle atrophy‐related ubiquitin E3 ligases Atrogin‐1 and MuRF1 and markedly reduced the expression of inflammasome‐related proteins (NLRP3, cleaved caspase‐1 and NT‐GSDMD) and pro‐inflammatory cytokines (IL‐1β and IL‐18), confirming its anti‐inflammatory and muscle‐protective effects.

Sepsis‐induced muscle wasting is closely associated with excessive inflammation mediated by NLRP3 inflammasome activation [19]. In the CLP‐induced sepsis mouse model, treatment with M12‐Liposome@MCC950 NPs significantly alleviated weight loss, muscle atrophy and functional impairment. Histological analysis showed that the NPs improved skeletal muscle fibre structure and significantly increased muscle fibre CSA. Western blot analysis further demonstrated that M12‐Liposome@MCC950 NPs effectively suppressed the expression of UPP‐related proteins Atrogin‐1 and MuRF1, indicating their ability to mitigate sepsis‐induced muscle atrophy via modulation of the ubiquitin–proteasome pathway. In both C2C12 cells and septic skeletal muscle tissues, sepsis markedly upregulated key inflammasome components (NLRP3, ASC, cleaved caspase‐1, IL‐1β and IL‐18), whereas M12‐Liposome@MCC950 NPs significantly inhibited their expression, confirming the potent inhibitory effect of the NPs on NLRP3 inflammasome activation.

Considering the hepatotoxicity reported in clinical studies of MCC950, we conducted comprehensive safety evaluations. Histological and biochemical analyses revealed no pathological damage in major organs (heart, liver, spleen, lung and kidney), and liver and kidney function markers (ALT, AST, CRE and BUN) remained within normal ranges. These findings indicate favourable biocompatibility and safety of the nanocarrier, supporting its further clinical translation.

## 4 Limitations and Future Perspectives

Despite the encouraging outcomes of this study, some limitations remain. First, although the therapeutic efficacy of M12‐Liposome@MCC950 NPs was demonstrated in animal models, further pharmacokinetic and biodistribution studies are needed to optimize the dosing regimen. Second, the potential immunomodulatory consequences of long‐term NLRP3 inhibition warrant further investigation. Finally, rigorous validation in larger animal models is required prior to clinical application to ensure safety and efficacy.

## 5 Conclusion

In conclusion, we successfully constructed a skeletal muscle‐targeted delivery system—M12‐Liposome@MCC950, which significantly enhanced MCC950 accumulation in skeletal muscle tissues, reduced systemic toxicity and ameliorated sepsis‐induced skeletal muscle inflammation and atrophy by inhibiting NLRP3 inflammasome activation and UPP‐mediated protein degradation. This study provides a new strategy for applying NLRP3 inhibitors in the treatment of SIM and offers theoretical support for skeletal muscle‐targeted nanodrug delivery systems, demonstrating great potential for clinical translation.

## Funding

This study was supported by grants from the National Natural Science Foundation of China (Nos. 82002101, 82002096), Hubei Provincial Natural Science Foundation of China (No. 2023AFB825) and Tongji Hospital, Tongji Medical College, Huazhong University of Science and Technology (No. 2023A15).

## Consent

The authors have nothing to report.

## Conflicts of Interest

The authors declare no conflicts of interest.

## Supporting information


**Table S1:** Detailed antibody information.

## Data Availability

Data sharing does not apply to this article as no new data were created or analysed in this study.
