# Supplementary material for: MCC950‐Loaded M12‐Liposome Nanoparticles for Targeted Inhibition of NLRP3 Inflammasome in Sepsis‐Induced Muscle Atrophy
Source: J Cachexia Sarcopenia Muscle. 2026 Apr 15;17(2):e70285. doi: 10.1002/jcsm.70285 (PMC13080895; doi:10.1002/jcsm.70285)
Supplement: Supplementary file 1 — Table S1: Detailed antibody information. [file JCSM-17-e70285-s001.docx]

| **Target Protein** | **Host Species** | **Catalog No.** | **Manufacturer** |
| --- | --- | --- | --- |
| **Primary Antibodies** |  |  |  |
| NLRP3 | Rabbit | A24294 | Abclonal, China |
| Caspase-1 | Rabbit | A25308 | Abclonal, China |
| MuRF1 (Trim63) | Rabbit | A3101 | Abclonal, China |
| Atrogin-1 (FBXO32) | Rabbit | Ab74023 | Abcam, UK |
| GSDMD | Rabbit | A28573 | Abclonal, China |
| IL-1β | Rabbit | A22257 | Abclonal, China |
| IL-18 | Rabbit | A20473 | Abclonal, China |
| GAPDH | Rabbit | P60037 | Abmart, China |
| **Secondary Antibodies** |  |  |  |
| HRP conjugated |  | G3431 | Seville Biotechnology |

Table 1. Detailed antibody information.
